# Challenges in Diagnosis and Management of Pneumoperitoneum Associated with Pneumatosis Cystoides Intestinalis in Children: A Systematic Review

**DOI:** 10.3390/jcm14186479

**Published:** 2025-09-14

**Authors:** Christina Siouli, Konstantina Dimopoulou, Dimitra Dimopoulou, Aggeliki Krikri, Natalia Kelaidi, Nikolaos Zavras, Anastasia Dimopoulou

**Affiliations:** 1 Department of Pediatric Surgery, Children’s General Hospital “Aghia Sophia”, 115 27 Athens, Greece; s.kristi1711@gmail.com (C.S.); akrikri@gmail.com (A.K.); kelaidinatalia@gmail.com (N.K.); natasa_dimo@hotmail.com (A.D.); 2 Department of Gastroenterology, Korgialenio-Benakio Red Cross Hospital, 115 26 Athens, Greece; 3 Second Department of Pediatrics, Children’s General Hospital “Aglaia & Panagiotis Kyriakou”, School of Medicine, National and Kapodistrian University of Athens, 115 27 Athens, Greece; dimi_med@hotmail.com; 4 Department of Pediatric Surgery, Attikon University Hospital, School of Medicine, National and Kapodistrian University of Athens, 124 62 Athens, Greece; nzavras@med.uoa.gr

**Keywords:** pneumatosis cystoides intestinalis, pneumoperitoneum, pediatrics, children, management, treatment, pediatric surgery, diagnosis, gastrointestinal complications

## Abstract

**Background/Objectives:** Pneumatosis cystoides intestinalis (PCI) is a rare condition in children characterized by gas-filled cysts in the intestinal wall. The presence of pneumoperitoneum poses significant diagnostic and therapeutic challenges, often mimicking gastrointestinal perforation. This systematic review aims to summarize the existing evidence on PCI-associated pneumoperitoneum in children. **Methods:** A systematic literature search was conducted in PubMed/Medline for articles published from January 1972 to March 2025. Studies involving patients ≤16 years old with PCI-related pneumoperitoneum and providing information on clinical presentation, diagnosis, treatment, and outcomes were included. Data extraction and study selection were independently performed by two reviewers in accordance with PRISMA guidelines. **Results:** Out of 209 articles initially identified, 23 studies comprising 95 pediatric cases (age range: 4 months–14 years) were included. The majority had underlying conditions such as malignancies, autoimmune disorders, or gastrointestinal motility issues and were often treated with corticosteroids and/or immunosuppressants. Most cases were incidentally diagnosed through radiographic imaging. Conservative treatment (bowel rest, antibiotics, parenteral nutrition) was applied in 85% of cases, while surgical intervention occurred in 11%. Only three cases had confirmed intestinal perforation. All patients experienced resolution of PCI; however, 20 died due to unrelated underlying diseases. **Conclusion:** Despite the case heterogeneity of this review, PCI-related pneumoperitoneum in children is an uncommon but clinically important entity, particularly in immunocompromised patients, that may lead to misdiagnosis and unnecessary surgery. Conservative management is effective in most cases, and clinical findings should guide treatment decisions. Increased awareness among pediatricians and surgeons is crucial to avoid overtreatment.

## 1. Introduction

Pneumatosis cystoides intestinalis (PCI) is characterized by the presence of gas-filled cysts within the submucosa or subserosa of any part of the gastrointestinal tract [1]. While this entity is well recognized in premature neonates as an early radiologic sign of necrotizing enterocolitis due to bowel ischemia, PCI is extremely rare in older children [2]. In the pediatric population, it has been correlated with chemotherapy, immunosuppression, prolonged high-dose corticosteroid use, and underlying conditions such as inflammatory bowel disease, congenital heart disease and intestinal motility disorders [1,3,4]. However, its exact pathophysiology remains unclear. Most patients are asymptomatic, and the diagnosis is typically made by abdominal imaging (Χ-ray or CT), which reveals intramural gas and, in some cases, free intraperitoneal air. The latter may be misinterpreted as gastrointestinal perforation [5], leading to diagnostic uncertainty that can result in unnecessary surgical intervention. Management is generally conservative, including bowel rest and broad-spectrum antibiotics with or without total parenteral nutrition. This approach appears to offer comparable outcomes to surgical intervention in patients without clinical or laboratory signs of an acute abdomen [3].

In this systematic review, we aim to summarize the limited existing evidence on the pathogenesis, clinical features, diagnosis, and management of PCI-associated pneumoperitoneum in the pediatric population. The purpose of this study is to highlight the diagnostic and therapeutic challenges of this rare condition, enhance the current knowledge of its clinical course, and help identify the optimal treatment approach. Additionally, we present our own experience through a representative case of PCI-related pneumoperitoneum in a pediatric patient with underlying scleroderma.

## 2. Methods

### 2.1. Search Strategy

A systematic literature review was conducted by two independent reviewers using the PubMed/Medline electronic database, focusing on the pneumoperitoneum associated with PCI in children, covering the period from 1 January 1972 to 31 March 2025. The following search string was applied: (“pneumatosis cystoides intestinalis” OR “pneumatosis intestinalis”) AND (“child” OR “children” OR “pediatric” OR “infant” OR “adolescent”) AND (“pneumoperitoneum” OR “free air” OR “surgery” OR “management” OR “treatment” OR “diagnosis”).

Furthermore, the reference lists of eligible articles were manually reviewed to identify additional relevant studies. The literature search was restricted to the English language. All articles selected for inclusion were critically evaluated. Duplicates were removed using the Mendeley Reference Manager software (version 2.93.0, Elsevier Ltd., London, United Kingdom), followed by manual verification. This systematic review was conducted in accordance with the PRISMA guidelines; however, registration was not applicable, as the review did not include a meta-analysis or use unpublished data or clinical trial protocols.

The PRISMA 2020 checklist is available in Tables S1 and S2 of the Supplementary Materials.

### 2.2. Study Selection

In the first step of study selection, irrelevant studies were excluded following the title and abstract screening of the articles by two independent reviewers. Subsequently, the full texts of potentially eligible studies were screened and included only those meeting the following criteria: (1) pediatric patients (≤16 years old); (2) pneumoperitoneum associated with PCI; (3) original research articles, case reports, or case series, including both retrospective and prospective observational studies; (4) studies reporting on the incidence, clinical presentation, diagnostic methods, management strategies, and patient outcomes related to pneumoperitoneum; (5) articles written in English.

Review articles, systematic reviews, meta-analyses, editorials, expert opinions, and conference abstracts were excluded. Additionally, studies describing PCI without pneumoperitoneum, articles that did not specifically address pneumoperitoneum secondary to PCI, or those focusing on other complications were excluded. For studies that included both adult and pediatric patients, only the pediatric cases were extracted and analyzed for this review. After the first screening, title/abstract and full-text screening was performed by two researchers independently. Any disagreements regarding study eligibility between the two reviewers were resolved through discussion until consensus was reached. Inter-rater reliability for full-text screening was high (Cohen’s κ = 0.87).

### 2.3. Data Extracted

A standardized form was used for data extraction from the included studies. Data were extracted independently by the two reviewers. The following information was extracted and recorded in the database: first author, year of publication, age, gender, etiology, underlying disease, presenting symptoms, location of PCI, diagnostic method, treatment approach, outcome, and follow-up. Any disagreements in results between reviewers was resolved through discussion and consensus.

### 2.4. Quality Assessment

The quality of included studies was assessed using the Joanna Briggs Institute (JBI) critical appraisal checklists. Two reviewers independently evaluated each study, with discrepancies resolved through discussion with a senior reviewer. For studies where a single case was extracted, the JBI *Checklist for Case Reports* was applied. When two or more cases were analyzed, quality assessment depended on the approach: if cases were presented individually, the *Checklist for Case Reports* was used, whereas if analyzed collectively as part of the original case series, the *Checklist for Case Series* was applied. Each checklist item was rated as “yes”, “no”, “unclear”, or “not applicable”. Study quality was classified as good (>75% of items rated “yes”), moderate (50–75%), or poor (<50%). Articles rated as poor quality were critically examined for their potential impact on the overall conclusions of the review.

## 3. Results

**Figure 1:**
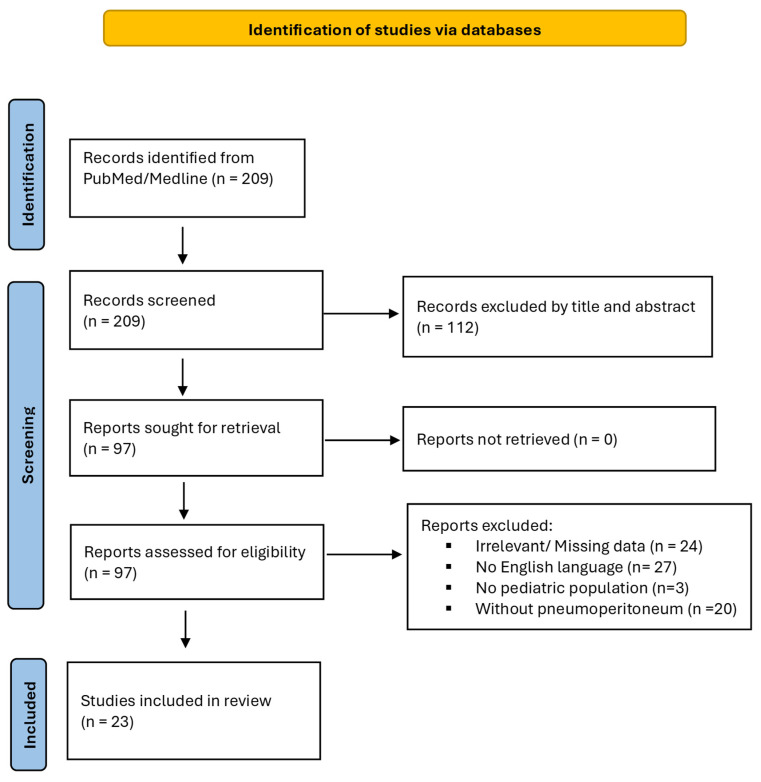
PRISMA 2020 flow chart illustrating the review process and article inclusion criteria.

A total of 209 articles were identified through the literature search. Following title and abstract screening, 112 articles were excluded. An additional 74 records were excluded after full-text review for not addressing pneumoperitoneum cases related to PCI (Figure 1). Ultimately, 23 published articles were eligible for inclusion, comprising 95 reported cases of pediatric patients with pneumoperitoneum related to PCI [1,2,3,4,6,7,8,9,10,11,12,13,14,15,16,17,18,19,20,21,22,23,24].

Table 1 summarizes the baseline characteristics, etiology of PCI, underlying diseases, clinical presentation, diagnostic methods, treatment approaches, and outcomes of the 95 patients included in this systematic review. Patient ages ranged from 4 months old to 14 years old. In total, 31 patients were male and 25 female, while the gender was not reported in 39 cases due to missing data in the original case reports, reflecting reporting inconsistencies (Table 1). Regarding the underlying diseases, 33 children were oncology patients of the oncology ward, 5 had hematologic disorders, and 2 had known autoimmune diseases. The remaining 55 had other underlying conditions such as biliary atresia, a1-antitrypsin deficiency, Down syndrome, cerebral palsy, abdominal tuberculosis, eosinophilic gastroenteritis, gastrointestinal dysmotility, and encephalitis (Table 1).

The most common presenting symptoms were abdominal pain, distention, and diarrhea, although 13 children were asymptomatic (Table 1). At the time of PCI diagnosis, most patients were receiving corticosteroids (n = 48) or combined corticosteroid and immunosuppressive therapy (n = 18). Only 1 patient was on immunosuppressive therapy alone, while in 28 cases, PCI was attributed to other medications such as antibiotics, or to the underlying disease. In almost all patients, PCI was diagnosed incidentally by abdominal Χ-ray or CT imaging, except for three children in whom abdominal radiograph was performed due to high clinical suspicion of bowel perforation. Regarding PCI location, in more than half of the patients, PCI was localized on the colon (Table 1).

**Figure 2:**
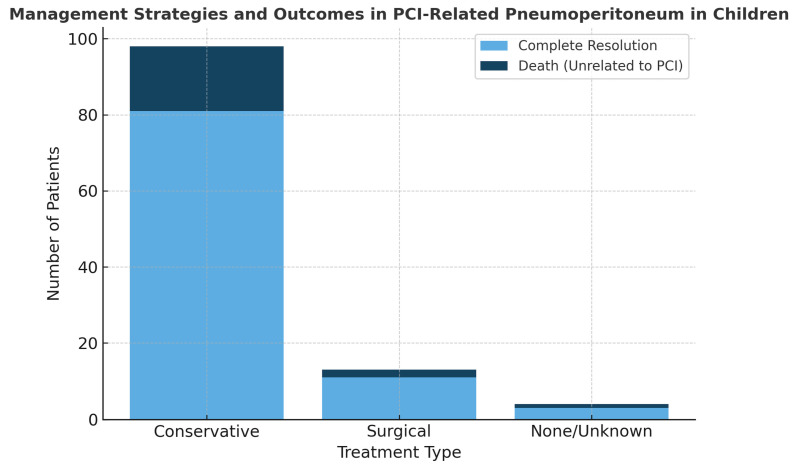
Management strategies and clinical outcomes in PCI-related pneumoperitoneum in children. Bar chart showing the distribution of patients according to treatment type (conservative, surgical, none/unknown). Each bar indicates the number of patients who achieved complete resolution (light blue) and the number of deaths unrelated to PCI (dark blue).

The vast majority of the patients (n = 81, 85.3%, 95% CI: 76.8–91.0%) were treated conservatively with bowel rest and intravenous antibiotics, with or without total parenteral nutrition. Of note, 11 patients (11.6%, 95% CI: 6.6–19.6%) underwent exploratory laparotomy, but bowel perforation was confirmed intraoperatively in only 3 patients (3.2%, 95% CI: 1.1–8.9%) In the remaining three patients, immunosuppressive therapy was discontinued, which contributed to the improvement and resolution of PCI. Finally, complete resolution of PCI was documented in all patients. However, 20 children later died, due to their underlying condition rather than PCI itself (Table 1, Figure 2).

### Quality Assessment

The methodological quality of the included studies, as assessed with the Joanna Briggs Institute (JBI) critical appraisal checklists, was overall good. Among the 12 case reports assessed, all achieved a score of 100%, corresponding to good quality (Table S3). Among the 11 case series (Table S4), 9 were rated as good quality, with scores ranging from 80% to 100%. Two case series (Jaffe et al. [17] and Galal et al. [15]) were rated as moderate quality (50%), mainly due to incomplete reporting of participant selection, demographics, and inclusion criteria. No study was classified as poor quality (<50%).

## 4. Discussion

PCI is extremely rare in the pediatric population, beyond the neonatal period. Pneumoperitoneum, though even less common, represents a significant complication of PCI that can further complicate diagnosis and management. The diagnosis of pneumoperitoneum, especially when associated with PCI, remains a challenge, and the optimal treatment is still under debate. To our knowledge, this is the first systematic review that sheds light on this uncommon complication of PCI focusing on the current diagnostic approaches, therapeutic strategies and treatment outcomes.

Although PCI is more frequently described in oncology patients as a complication of immunosuppression, our findings reveal that more than half of the children had non-oncologic underlying conditions, such as biliary atresia, α1-antitrypsin deficiency, Down syndrome, gastrointestinal dysmotility, or cerebral palsy. Only two previous case reports described PCI with pneumoperitoneum in children with autoimmune diseases. Given the extreme rarity of PCI-related pneumoperitoneum in pediatric autoimmune conditions, we present our own experience with a rare case in a child with scleroderma. This 15-year-old patient is described as an illustrative example and was not included in the pooled cohort of 95 patients from the systematic review.

**Figure 3:**
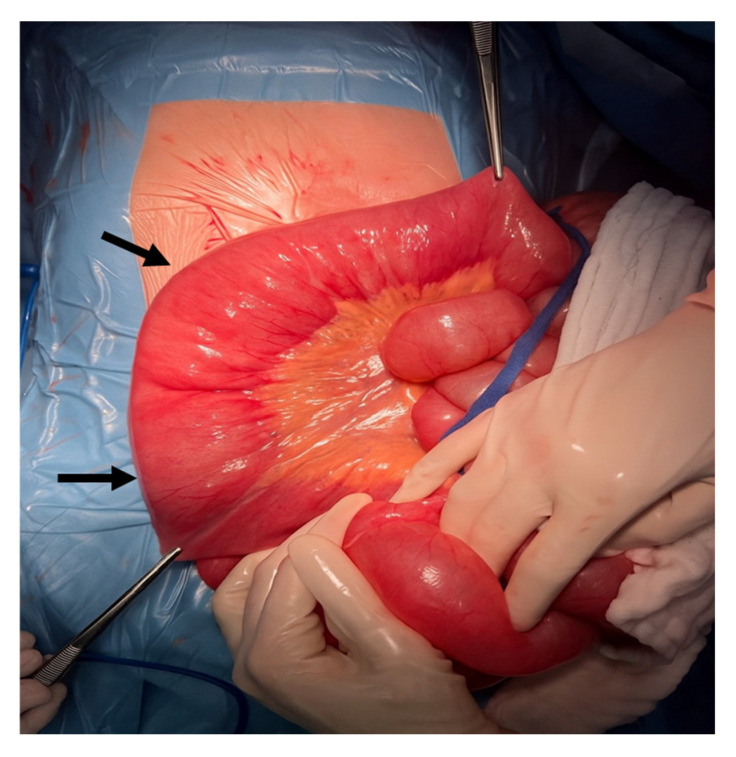
Intraoperative findings of markedly dilated ileus with numerous intramural gas-filled cysts (arrows) in a 15-year-old patient with scleroderma.

A 15-year-old female adolescent with a medical history of scleroderma treated with high-dose corticosteroids was hospitalized in the psychiatric ward due to hallucinations. Approximately one month after admission, a surgical consultation was requested for severe constipation and abdominal distention. Her vital signs and laboratory tests were unremarkable, with no fever, and abdominal examination revealed no tenderness, guarding, or other signs of peritonitis. Abdominal X-ray demonstrated a large volume of free intraperitoneal air, which was confirmed by abdominal CT. An exploratory laparotomy was performed immediately. Intraoperatively, no intestinal perforation was identified, however, a markedly dilated ileus with numerous intramural gas-filled cysts was observed (Figure 3). A full-thickness intestinal biopsy was obtained, which confirmed the diagnosis of PCI. Postoperatively, the patient was managed with bowel rest and total parenteral nutrition due to intestinal paresis and recurrent episodes of vomiting. Follow-up radiographs showed complete resolution of PCI. She was subsequently transferred to the pediatric ward for further assessment and management of her intestinal paresis. This case related to pediatric scleroderma adds to this very limited evidence, highlighting the rarity of this presentation. This observation expands the recognized clinical manifestations of PCI and emphasizes the need for greater clinical suspicion in non-oncologic children receiving immunosuppressive therapy.

The pathogenesis of PCI remains unclear; however, several theories have been proposed. In oncology patients, it is hypothesized that submucosal or subserosal cysts are formed as a result of mucosal injury caused by chemotherapy or radiation [9]. Similarly, in patients with inflammatory bowel disease, extended inflammation of the bowel mucosa may lead to PCI [4]. Another widely suggested theory is intestinal bacterial overgrowth, especially in immunocompromised patients. This facilitates gas accumulation in the submucosa due to increased mucosal permeability or direct mucosal damage [9]. Pneumoperitoneum in the setting of PCI is most commonly attributed to the rupture of subserosal cysts within the intestinal wall allowing gas to escape into the peritoneal cavity without bowel perforation [25]. This rupture of gas-filled cysts may be precipitated by conditions that increase intraluminal pressure, such as bowel obstruction or motility disorders [26]. Additionally, in immunocompromised patients, weakened intestinal wall layers may facilitate the translocation of gas into the peritoneal cavity [26]. Indeed, the majority of patients were on corticosteroids alone or in combination with immunosuppressive agents at the time of diagnosis. This observation supports the theory that corticosteroid-induced lymphopenia, can be associated with damage of the muscularis mucosae of the gastrointestinal tract, resulting in intraluminal gas passing to the submucosa layer [27]. Interestingly, almost all cases were diagnosed incidentally through imaging—most commonly via X-ray or CT. Only three children underwent imaging due to a high clinical suspicion of perforation. These findings indicate that PCI and its complications often have no clear symptoms, highlighting the need for thorough radiological evaluation, particularly in high-risk patients. Recent reports highlight the role of modern imaging modalities in differentiating benign from clinically worrisome PCI-related findings, with CT features such as bowel wall thickness and mesenteric stranding serving as useful predictors [25]. In addition, complementary techniques such as abdominal ultrasound and MRI using gradient-echo sequences have been described to aid in detecting intramural gas without radiation exposure, an aspect of particular importance in the pediatric population [26,28,29]. Although PCI can affect any part of the bowel, to any extent, and with various amounts of pneumoperitoneum with or without portal venous gas, there is no evidence that these features correlate with disease severity or indicate the need for surgical intervention [5,30].

While pneumoperitoneum is a classic surgical emergency, our review indicates that in the setting of PCI, surgery may not always be necessary. Approximately 85% of patients were managed conservatively with bowel rest, broad-spectrum antibiotics, with or without parenteral nutrition. Surgical intervention was performed in about 11% of cases, but bowel perforation was confirmed in only a small subset of those cases. Since this entity is mostly diagnosed incidentally and children are usually asymptomatic or present with non-specific symptomatology, it is crucial to have strong indications before proceeding to surgery. These findings suggest that clinical stability, rather than imaging results alone, should guide the management strategy.

**Figure 4:**
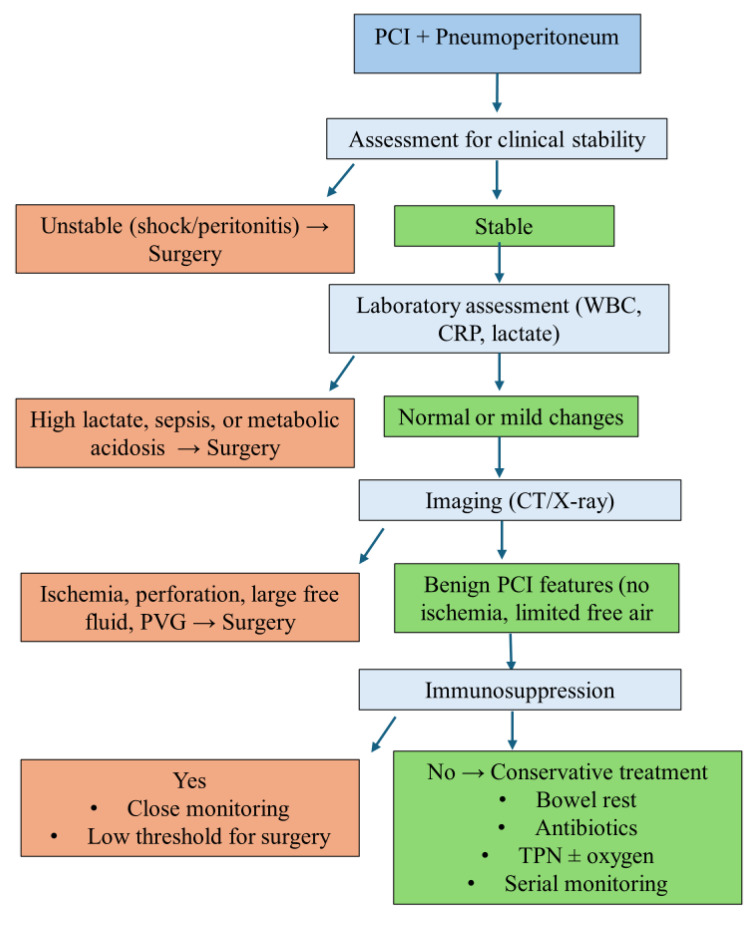
Proposed decision-making algorithm for the management of pneumoperitoneum associated with pneumatosis cystoides intestinalis in children. PCI, pneumatosis cystoides intestinalis; CRP, C-reactive protein; WBC, white blood cell count; CT, computed tomography; X-ray, radiograph; PVG, portal venous gas; TPN, total parenteral nutrition.

Recent adult data reinforce the safety of conservative management even in PCI complicated by pneumoperitoneum. Than et al. described four adult cases of benign PCI with pneumoperitoneum, three of which were successfully managed without surgery [31]. In a large systematic review of 1673 patients, pneumoperitoneum was reported in nearly 18% of cases, with conservative treatment applied in almost half, further supporting a watch-and-wait approach in selected patients [32]. Similarly, Tropeano et al. emphasized that even in the presence of pneumoperitoneum or portal venous gas, surgery should not be automatically pursued in stable patients, but instead management should be individualized [33]. It is important to recognize that clinical and laboratory findings in immunocompromised patients or patients receiving high doses of corticosteroids may be misleading [23]. A thorough clinical evaluation is essential, particularly because signs of peritonitis may be subtle or absent in immunocompromised children. In those patients, the decision to intervene surgically is the most challenging. This is further complicated by the fact that no radiological pattern, such as extent, location, or volume of gas, has been associated with disease severity or the need for surgery [30]. A proposed decision-making algorithm is presented in Figure 4, summarizing key clinical, laboratory, and imaging factors that can guide management of PCI with pneumoperitoneum.

This systematic review is the first to investigate pneumoperitoneum associated with PCI in the pediatric population, highlighting diagnostic and management strategies based on a synthesis of 95 cases across 23 studies. The inclusion of a representative case further enhances the clinical relevance of our findings. However, this review has several limitations. First, the heterogeneity of the included case reports with incomplete or inconsistent data could result in variability in the findings. The methodological quality of the included studies, as assessed with the JBI critical appraisal checklists, was overall good, but some reports lacked complete demographic information, standardized diagnostic methods, or long-term follow-up, and several case series relied only on descriptive analyses (Tables S3 and S4). These factors introduce a potential risk of bias that needs to be considered when interpreting the results. The retrospective nature and the absence of standardized diagnostic or therapeutic criteria also limit the ability to establish causality. Moreover, publication bias and underreporting of asymptomatic or conservatively managed cases may have influenced the findings. In conclusion, PCI-related pneumoperitoneum in children is a rare but important diagnostic entity, particularly in immunocompromised and corticosteroid-treated patients. Pediatricians and surgeons should be aware of the existence of pneumoperitoneum in the setting of PCI and must rely mostly on careful clinical examination. Surgical intervention should be performed in cases with indisputable evidence of bowel perforation, since the conservative management could be as effective as surgery and may be associated with shorter hospital stay. Future prospective multicenter studies and international registry efforts are needed to establish clearer criteria for when surgical intervention is truly necessary in order to reduce reporting inconsistencies and identify predictors of outcome. Standardized data collection would also decrease heterogeneity and improve comparability across studies. Such initiatives could guide the development of evidence-based management protocols for the diagnosis and treatment of PCI-related pneumoperitoneum in children.

## Figures and Tables

**Table 1:** Summary of reported pediatric cases of pneumatosis cystoides intestinalis with pneumoperitoneum. Legend/Data Dictionary: Individual case reports are listed separately; case series report the total number of included patients in parentheses. M/F: male/female. y.o.: years old. Underlying disease: baseline comorbidity or chronic condition (e.g., malignancy, congenital heart disease, autoimmune disease, gastrointestinal dysmotility). Etiology: suspected cause of PCI (e.g., corticosteroid therapy, chemotherapy, immunosuppression, infection, trauma, other medication). PCI location: anatomic site(s) of pneumatosis; multiple entries possible if more than one segment was affected: (SB, small bowel; TI, terminal ileum; AC, ascending colon; HF, hepatic flexure; TC, transverse colon; ICV, ileocecal valve). Diagnosis method confirming PCI and/or pneumoperitoneum (abdominal X-ray (AXR), chest X-ray (CXR), ultrasound (US), computed tomography (CT), intraoperative or autopsy findings. Treatment: classified as conservative (fasting, antibiotics, oxygen, total parenteral nutrition), surgical (laparotomy, resection, stoma), or none/unknown (no treatment reported or not administered). Outcome: clinical and/or radiological resolution of PCI-related pneumoperitoneum or death from underlying disease, unrelated to PCI. Follow-up: duration and findings as reported in the original publication. N/A: not available in the original report or not applicable for the specific variable.

Study (Year)	Patient No.	Gender	Age (y.o.)	Underlying Disease (No Patients)	Etiology	Symptoms (No Patients)	PCI Location (No Patients)	Diagnosis (No Patients)	Treatment (No Patients)	Outcome (No Patients)	Follow-Up (No Patients)
Alp et al. (2010) [7]	1	N/A	8	Abdominal TB	Abdominal TB	Distension, tenderness	SB, gastritis, duodenitis	AXR, U/S, CT, endoscopy	Conservative	Died (unknown cause)	3 weeks: little improvement
Schuster et al. (2017) [4]	1	M	14	Fanconi anemia	Corticosteroids for GvHD, incomplete atrophic desmosis of colon	Bloody diarrhea, pain, anemia	TI, colon (sparing sigmoid + rectum)	CT	Surgical	Ostomy reversal after 4 months	1 year: resolved
Berard et al. (2010) [11]	1	F	8	Juvenile dermatomyositis	Corticosteroids, MTX	Asymptomatic	Colon	CXR, CT	Conservative	Resolved	6 weeks: resolved
Chang et al. (2015) [13]	1	F	13	Granulomatosis with polyangiitis	Corticosteroids, MTX	Asymptomatic	SB, colon	AXR, CT	Conservative	Resolved	17 months: resolved
Kim et al. (2021) [19]	1	F	1	B-ALL	Blinatumomab	Constipation	AC, HF	CT	Conservative	Resolved	1 year: resolved
McCarville et al. (2008) [20]	16	11M/3F	1–13	AML (5) ALL (5) Sickle cell anemia (2) Neuroblastoma (1) Myeloblastoma (1)	Corticosteroids	Emesis (2) Pain/diarrhea (9) Asymptomatic (3)	Colon	AXR, CT	Conservative	Resolved	N/A
Ryan et al. (2020) [22]	8	4M/4F	1–7	Hepatoblastoma (3) Biliary atresia (4) Alpha-1 antitrypsin deficiency (1)	Immunosuppressive therapy post-transplant, corticosteroids	Diarrhea (5) Bloody stools (1) Asymptomatic (2)	Cecum, AC, TC	US, CT	Conservative	Resolved	N/A
Naiditch et al. (2010) [21]	1	M	3	ALL	Immunosuppressive therapy for GvHD, corticosteroids, antibiotics	Pain, bilious emesis, fever	SB	CXR, CT	Conservative	Resolved	N/A
Jaffe et al. (1972) [17]	6	4M/2F	1–5	ALL	Chemotherapy, corticosteroids, ACTH, blood transfusions, antibiotics	Tenderness/pain (3) Left pneumothorax, pleural effusion, lobar pneumonia (1) Distention (1) Asymptomatic (1)	Colon (1) Colon + rectum (1) Cecum (1) AC (1) Ileus (1) ICV, cecum, AC (1)	AXR (3) Autopsy (3)	Conservative (4) N/A (2)	Resolved (1) Died (5)	Died due to sepsis 5 years later (1) Autopsy showed PCI (5); cause of death was leukemia and sepsis (4) and electrolyte imbalance (1)
Aygunes et al. (2023) [9]	1	F	4	AML	Chemotherapy	Fever, pain	Colon	AXR, CT	Conservative	Resolved (21 days)	Inpatient for transplant
D’Agostino et al. (2000) [14]	1	M	3	Down syndrome	N/A	Distension, emesis	Jejunum, ileum, colon	AXR	Surgical	Resolved	1 year: resolved
Tang et al. (1998) [2]	5	N/A	4 mo–11 y.o.	Combined immunodeficiency	GvHD, corticosteroids	Diarrhea ± blood, distension, pain, fever (4) Asymptomatic (1)	N/A	AXR, US	Conservative	Resolved (4) Died (1)	2–3.5 weeks: resolved
Kim et al. (2005) [18]	1	F	9	TBI	N/A	Distension, bloody diarrhea	N/A	AXR	Conservative	Resolved	10 days: resolved
Hochwald et al. (2007) [16]	1	F	1.5	Rhabdomyosarcoma	N/A	Diarrhea, pain, distention	Colon	CT	Conservative	Resolved	N/A
Chan et al. (2010) [12]	1	M	2	Nephrotic syndrome	Corticosteroids	Diarrhea, distension,	Colon, rectum	CXR, CT	Conservative	Resolved	N/A
Awad et al. (2017) [8]	5	2M/3F	5–9	Cerebral palsy	No corticosteroids or immunosuppression	Pain, distention	Colon	AXR	Surgical (4) Conservative (1)	Resolved	Recurrence (4): conservative management
Yeager et al. (1987) [24]	1	M	5	Aplastic anemia/BMT	Corticosteroids	Tenderness	Colon	AXR	Conservative	Resolved	220 days: resolved
Galal et al. (1981) [15]	2	2F	4–6	ALL	Corticosteroids, chemotherapy	Maxillary sinusitis (1) Cough, thoracic tenderness, distention (1)	AC, TC	AXR, CXR	Conservative	Resolved	N/A
Ade-Ajayi et al. (2002) [6]	3	1M/2F	11	ALL (1) AML (2)	Corticosteroids	Pain, diarrhea, distension	N/A	AXR	Conservative	Resolved	Died due to leukemia
Wallace et al. (2021) [23]	15	N/A	N/A	GvHD	Corticosteroids	Distention (11) Asymptomatic (4)	N/A	AXR	Conservative	Resolved	100 days: died (8) due to other reasons
Kurbegov et al. (2001) [3]	8	N/A	N/A	Tissue transplant, CHD, GI dysmotility	GvHD, Colitis, Ischemia	Distension, pain. diarrhea, fever, emesis	N/A	AXR	Conservative (5) Surgical (2) None (1)	Resolved	N/A
Bailey et al. (2022) [10]	8	N/A	N/A	Oncology patients	Corticosteroids	Pain, distention	N/A	AXR	Conservative (6) Surgical (2)	Resolved	N/A
Galea et al. (2016) [1]	7	4M/3F	5–14	Developmental delay, ALL, encephalitis, eosinophilic gastroenteritis	N/A	Chest pain, seizure, gastroenteritis	Cecum, AC	CT	Conservative (6) Surgical (1)	Resolved	Died (1) due to encephalitis

No, number; N/A, not applicable; M, male; F, female; y.o., years old; TB, tuberculosis; ALL, Acute Lymphoblastic Leukemia; AML, Acute Myeloid Leukemia; B-ALL, B-cell Acute Lymphoblastic Leukemia; TBI, Traumatic Brain Injury; BMT, Bone Marrow Transplant; CHD, congenital heart disease; GI, gastrointestinal; GvHD, Graft-versus-Host Disease; MTX, methotrexate; ACTH, Adrenocorticotropic Hormone; PCI, pneumatosis cystoides intestinalis; SB, small bowel; TI, terminal ileum; AC, ascending colon; HF, hepatic flexure; TC, transverse colon; ICV, ileocecal valve; U/S, ultrasound; CT, computed tomography; AXR, abdominal X-ray; CXR, chest X-ray.

## Data Availability

The data underlying this article will be shared on reasonable request to the corresponding author.

## Supplementary Materials

The following supporting information can be downloaded at: https://www.mdpi.com/article/10.3390/jcm14186479/s1, Table S1: Preferred Reporting Items for Systematic Reviews and Meta-Analyses (PRISMA) 2020 abstract checklist; Table S2: Preferred Reporting Items for Systematic Reviews and Meta-Analyses (PRISMA) 2020 checklist; Table S3: Quality assessment of the included studies using the Joanna Briggs Institute critical appraisal *Checklist for Case Reports*; Table S4: Quality assessment of the included studies using the Joanna Briggs Institute critical appraisal *Checklist for Case Series*.

## Abbreviations

PCI	Pneumatosis cystoides intestinalis
No	Number
N/A	Not Applicable
M	Male
F	Female
y.o.	Years Old
TB	Tuberculosis
ALL	Acute Lymphoblastic Leukemia
AML	Acute Myeloid Leukemia
TBI	Traumatic Brain Injury
BMT	Bone Marrow Transplant
GvHD	Graft-versus-Host Disease
MTX	Methotrexate
ACTH	Adrenocorticotropic Hormone
SB	Small Bowel
TI	Terminal Ileum
AC	Ascending Colon
HF	Hepatic Flexure
TC	Transverse Colon
ICV	Ileocecal Valve
U/S	Ultrasound
CT	Computed Tomography
CXR	Chest X-ray
JBI	Joanna Briggs Institute
