# Supplementary material for: Challenges in Diagnosis and Management of Pneumoperitoneum Associated with Pneumatosis Cystoides Intestinalis in Children: A Systematic Review
Source: J Clin Med. 2025 Sep 14;14(18):6479. doi: 10.3390/jcm14186479 (PMC12470710; doi:10.3390/jcm14186479)
Supplement: Supplementary file 1 [file jcm-14-06479-s001.zip › jcm-3808490-supplementary.pdf]

Supplementary Table S1: Preferred Reporting Items for Systematic Reviews and Meta-Analyses (PRISMA) 2020 abstract checklist.

	Item #	Checklist item	Reported (Yes/No)
**TITLE**			
Title	1	Identify the report as a systematic review.	Yes
**BACKGROUND**			
Objectives	2	Provide an explicit statement of the main objective(s) or question(s) the review addresses.	Yes
**METHODS**			
Eligibility criteria	3	Specify the inclusion and exclusion criteria for the review.	Yes
Information sources	4	Specify the information sources (e.g. databases, registers) used to identify studies and the date when each was last searched.	Yes
Risk of bias	5	Specify the methods used to assess risk of bias in the included studies.	NA
Synthesis of results	6	Specify the methods used to present and synthesise results.	NA
**RESULTS**			
Included studies	7	Give the total number of included studies and participants and summarise relevant characteristics of studies.	Yes
Synthesis of results	8	Present results for main outcomes, preferably indicating the number of included studies and participants for each. If meta-analysis was done, report the summary estimate and confidence/credible interval. If comparing groups, indicate the direction of the effect (i.e. which group is favoured).	Yes
**DISCUSSION**			
Limitations of evidence	9	Provide a brief summary of the limitations of the evidence included in the review (e.g. study risk of bias, inconsistency and imprecision).	Yes
Interpretation	10	Provide a general interpretation of the results and important implications.	Yes
**OTHER**			
Funding	11	Specify the primary source of funding for the review.	Yes
Registration	12	Provide the register name and registration number.	NA

NA: Not applicable

Supplementary Table S2: Preferred Reporting Items for Systematic Reviews and Meta-Analyses (PRISMA) 2020 checklist.

Section and Topic	Item #	Checklist item	Location where item is reported
**TITLE**			
Title	1	Identify the report as a systematic review.	Title
**ABSTRACT**			
Abstract	2	See the PRISMA 2020 for Abstracts checklist.	Abstract, Table S1
**INTRODUCTION – PART 1**			
Rationale	3	Describe the rationale for the review in the context of existing knowledge.	Introduction – 1st paragraph
Objectives	4	Provide an explicit statement of the objective(s) or question(s) the review addresses.	Introduction – 2nd paragraph
**METHODS – PART 2**			
Eligibility criteria	5	Specify the inclusion and exclusion criteria for the review and how studies were grouped for the syntheses.	Methods, Study Selection
Information sources	6	Specify all databases, registers, websites, organisations, reference lists and other sources searched or consulted to identify studies. Specify the date when each source was last searched or consulted.	Methods, Search Strategy
Search strategy	7	Present the full search strategies for all databases, registers and websites, including any filters and limits used.	Methods, Search Strategy
Selection process	8	Specify the methods used to decide whether a study met the inclusion criteria of the review, including how many reviewers screened each record and each report retrieved, whether they worked independently, and if applicable, details of automation tools used in the process.	Methods, Study Selection
Data collection process	9	Specify the methods used to collect data from reports, including how many reviewers collected data from each report, whether they worked independently, any processes for obtaining or confirming data from study investigators, and if applicable, details of automation tools used in the process.	Methods, Data extracted
Data items	10a	List and define all outcomes for which data were sought. Specify whether all results that were compatible with each outcome domain in each study were sought (e.g. for all measures, time points, analyses), and if not, the methods used to decide which results to collect.	Methods, Data extracted

Section and Topic	Item #	Checklist item	Location where item is reported
	10b	List and define all other variables for which data were sought (e.g. participant and intervention characteristics, funding sources). Describe any assumptions made about any missing or unclear information.	Methods, Data extracted
Study risk of bias assessment	11	Specify the methods used to assess risk of bias in the included studies, including details of the tool(s) used, how many reviewers assessed each study and whether they worked independently, and if applicable, details of automation tools used in the process.	Methods, Quality Assessment
Effect measures	12	Specify for each outcome the effect measure(s) (e.g. risk ratio, mean difference) used in the synthesis or presentation of results.	NA
Synthesis methods	13a	Describe the processes used to decide which studies were eligible for each synthesis (e.g. tabulating the study intervention characteristics and comparing against the planned groups for each synthesis (item #5)).	NA
	13b	Describe any methods required to prepare the data for presentation or synthesis, such as handling of missing summary statistics, or data conversions.	NA
	13c	Describe any methods used to tabulate or visually display results of individual studies and syntheses.	NA
	13d	Describe any methods used to synthesize results and provide a rationale for the choice(s). If meta-analysis was performed, describe the model(s), method(s) to identify the presence and extent of statistical heterogeneity, and software package(s) used.	NA
	13e	Describe any methods used to explore possible causes of heterogeneity among study results (e.g. subgroup analysis, meta-regression).	NA
	13f	Describe any sensitivity analyses conducted to assess robustness of the synthesized results.	NA
Reporting bias assessment	14	Describe any methods used to assess risk of bias due to missing results in a synthesis (arising from reporting biases).	NA
Certainty assessment	15	Describe any methods used to assess certainty (or confidence) in the body of evidence for an outcome.	NA
**RESULTS – PART 3**			
Study selection	16a	Describe the results of the search and selection process, from the number of records identified in the search to the number of studies included in the review, ideally using a flow diagram.	Figure 1, Results -1st paragraph
	16b	Cite studies that might appear to meet the inclusion criteria, but which were excluded, and explain why they were excluded.	Figure 1, Results -1st paragraph
Study characteristics	17	Cite each included study and present its characteristics.	Table 1, Figure 2

Section and Topic	Item #	Checklist item	Location where item is reported
Risk of bias in studies	18	Present assessments of risk of bias for each included study.	Methods, Quality Assessment
Results of individual studies	19	For all outcomes, present, for each study: (a) summary statistics for each group (where appropriate) and (b) an effect estimate and its precision (e.g. confidence/credible interval), ideally using structured tables or plots.	Table 1, Figure 2, Results – 2nd-4th paragraph
Results of syntheses	20a	For each synthesis, briefly summarise the characteristics and risk of bias among contributing studies.	NA
	20b	Present results of all statistical syntheses conducted. If meta-analysis was done, present for each the summary estimate and its precision (e.g. confidence/credible interval) and measures of statistical heterogeneity. If comparing groups, describe the direction of the effect.	Table 1, Figure 2, Results – 2nd-4th paragraph
	20c	Present results of all investigations of possible causes of heterogeneity among study results.	NA
	20d	Present results of all sensitivity analyses conducted to assess the robustness of the synthesized results.	NA
Reporting biases	21	Present assessments of risk of bias due to missing results (arising from reporting biases) for each synthesis assessed.	NA
Certainty of evidence	22	Present assessments of certainty (or confidence) in the body of evidence for each outcome assessed.	NA
**DISCUSSION – PART 4**			
Discussion	23a	Provide a general interpretation of the results in the context of other evidence.	Discussion – 1st -7th paragraph
	23b	Discuss any limitations of the evidence included in the review.	Discussion – 8th paragraph
	23c	Discuss any limitations of the review processes used.	Discussion – 8th paragraph
	23d	Discuss implications of the results for practice, policy, and future research.	Discussion – 9th paragraph
**OTHER INFORMATION**			
Registration and protocol	24a	Provide registration information for the review, including register name and registration number, or state that the review was not registered.	NA

Section and Topic	Item #	Checklist item	Location where item is reported
	24b	Indicate where the review protocol can be accessed, or state that a protocol was not prepared.	NA
	24c	Describe and explain any amendments to information provided at registration or in the protocol.	NA
Support	25	Describe sources of financial or non-financial support for the review, and the role of the funders or sponsors in the review.	Funding
Competing interests	26	Declare any competing interests of review authors.	Conflicts of Interest
Availability of data, code and other materials	27	Report which of the following are publicly available and where they can be found: template data collection forms; data extracted from included studies; data used for all analyses; analytic code; any other materials used in the review.	Data Availability Statement

NA: Not applicable

Table S3. Quality assessment of the included studies using the Joanna Briggs Institute Critical Appraisal Checklist for Case Reports.

STUDY	Q1	Q2	Q3	Q4	Q5	Q6	Q7	Q8	% YES	STUDY QUALITY
Alp et al. (2010) [7]	Y	Y	Y	Y	Y	Y	Y	Y	100	GOOD
Schuster et al. (2017) [4]	Y	Y	Y	Y	Y	Y	Y	Y	100	GOOD
Berard et al. (2010) [11]	Y	Y	Y	Y	Y	Y	Y	Y	100	GOOD
Chang et al. (2015) [13]	Y	Y	Y	Y	Y	Y	Y	Y	100	GOOD
Kim et al. (2021) [19]	Y	Y	Y	Y	Y	Y	Y	Y	100	GOOD
Naiditch et al. (2010) [21]	Y	Y	Y	Y	Y	Y	Y	Y	100	GOOD
Aygunes et al. (2023) [9]	Y	Y	Y	Y	Y	Y	Y	Y	100	GOOD
D'Agostino et al. (2000) [14]	Y	Y	Y	Y	Y	Y	Y	Y	100	GOOD
Kim et al. (2005) [18]	Y	Y	Y	Y	Y	Y	Y	Y	100	GOOD
Hochwald et al. (2007) [16]	Y	Y	Y	Y	Y	Y	Y	Y	100	GOOD
Chan et al. (2010) [12]	Y	Y	Y	Y	Y	Y	Y	Y	100	GOOD
Yeager et al. (1987) [24]	Y	Y	Y	Y	Y	Y	Y	Y	100	GOOD

QUESTIONS

Q1. Were the patient's demographic characteristics clearly described?

Q2. Was the patient's history clearly described and presented as a timeline?

Q3. Was the current clinical condition of the patient on presentation clearly described?

Q4.Were diagnostic tests or assessment methods and the results clearly described?

Q5. Was the intervention(s) or treatment procedure(s) clearly described?

Q6. Was the post-intervention clinical condition clearly described?

Q7. Were adverse events (harms) or unanticipated events identified and described?

Q8. Does the case report provide takeaway lessons?

FOOTNOTES

'Y' indicates yes, 'N' indicates no, and 'U' indicates unclear.

Table S4. Quality assessment of the included studies using the Joanna Briggs Institute Critical Appraisal Checklist for Case Series.

STUDY	Q1	Q2	Q3	Q4	Q5	Q6	Q7	Q8	Q9	Q10	% YES	STUDY QUALITY
McCarville et al. (2008) [20]	Y	Y	Y	Y	Y	Y	Y	Y	Y	Y	100	GOOD
Ryan et al. (2020) [22]	Y	Y	Y	U	U	Y	Y	Y	Y	Y	100	GOOD
Jaffe et al. (1972) [17]	U	U	Y	U	U	Y	Y	Y	Y	N	50	MODERATE
Tang et al. (1998) [2]	Y	Y	Y	Y	Y	U	Y	Y	Y	N	80	GOOD
Awad et al. (2017) [8]	Y	Y	Y	Y	Y	Y	Y	Y	Y	N	90	GOOD
Galal et al. (1981) [15]	N	U	Y	U	U	Y	Y	Y	Y	N	50	MODERATE
Ade-Ajayi et al. (2002) [6]	Y	Y	Y	Y	Y	Y	Y	Y	Y	N	90	GOOD
Wallace et al. (2021) [23]	Y	Y	Y	Y	Y	Y	Y	Y	Y	Y	100	GOOD
Kurbegov et al. (2001) [3]	Y	Y	Y	Y	Y	Y	Y	Y	Y	Y	100	GOOD
Bailey et al. (2022) [10]	Y	Y	Y	Y	Y	Y	Y	Y	Y	Y	100	GOOD
Galea et al. (2016) [1]	Y	Y	Y	Y	Y	Y	Y	Y	Y	N	90	GOOD

QUESTIONS

Q1. Were there clear criteria for inclusion in the case series?

Q2. Was the condition measured in a standard, reliable way for all participants included in the case series?

Q3. Were valid methods used for identification of the condition for all participants included in the case series?

Q4. Did the case series have consecutive inclusion of participants?

Q5. Did the case series have complete inclusion of participants?

Q6. Was there clear reporting of the demographics of the participants in the study?

Q7. Was there clear reporting of clinical information of the participants?

Q8. Were the outcomes or follow up results of cases clearly reported?

Q9. Was there clear reporting of the presenting site(s)/clinic(s) demographic information?

Q10. Was statistical analysis appropriate?

FOOTNOTES

'Y' indicates yes, 'N' indicates no, and 'U' indicates unclear.
